# What do people die from? The challenges of measuring disease burden posed by multi-morbidity

**DOI:** 10.1186/s13584-015-0047-2

**Published:** 2015-10-05

**Authors:** Martin McKee

**Affiliations:** European Public Health, London School of Hygiene and Tropical Medicine, 15-17 Tavistock Place, London, WC1H 9SH UK

## Abstract

Determining the precise cause of death of an individual has become increasingly difficult as life prolonging treatments mean that many people may die with multiple disorders, any of which, individually or in concert, might have killed them. Yet this problem has been recognised from the very earliest attempts to develop classifications of diseases. This paper traces the evolution of thinking on death certification and disease classification, describing what has been achieved but also identifying what more needs to be done to advance research and inform policy.

## Background

In the accompanying paper Goldberger and colleagues illustrate clearly the challenges of making international comparisons of disease burden [1]. They seek to explain the observation that reported death rates from cardiovascular disease in Israel are low but those from diabetes and renal failure are high. Taking advantage of data on multiple causes of death, they conclude that the seemingly high rates of diabetes and renal failure may in part be attributed to coding practices and some of these deaths might be attributed to cardiovascular disease elsewhere. However this does not provide a complete explanation, indicating the need for an appropriate policy response.

## The emergence of disease classifications

Their paper addresses a question that is superficially simple but, in reality, very complex [2]. What do people die from? Historically, if physicians stated any cause of death at all, it would be arbitrary, reflecting their often idiosyncratic habits or the tradition of the medical school in which they trained. By the eighteenth century several physicians who were also botanists, such as Linnaeus, sought to describe and then classify causes, just as they had done with plants. Yet, given the very limited contemporary understanding of pathology and thus the inability to link many deaths to particular organs, the results were based on little more than symptoms or mode of death. By the mid-nineteenth century William Farr in England [3] and Marc d’Espine in Geneva [4] had provided the basis for a classification, albeit somewhat basic. Thus, Farr’s groups included endemic and epidemic diseases, typically caused by infections, sporadic diseases, including many non-communicable diseases, and violence. Within these categories there was an attempt, albeit imperfect, to link causes to organs, to the extent that this was possible given the rudimentary knowledge of body systems at the time. Although their classifications were used by their compatriots, they spread little further, reflecting a view that classification of disease may be impossible because of the diversity in terminology used by physicians.

Yet some were not discouraged. One was Dr Jacques Bertillon, a French physician who would go on to develop the first recognisably modern classification of causes of death, with 14 chapters listed mostly according to body system [5]. Bertillon is rightly celebrated for his innovation, paving the way for modern epidemiology. However, as he was preparing his system in 1893, his task was somewhat simpler than it would be today. In most cases, those who died did so from a single cause of death, often an injury or an infection. An extended and revised version of this system would be adopted in 1900 as the International Classification of Causes of Death (ICD), with the recommendation that it be revised every 10 years.

## Chronic disease and the rising burden of multi-morbidity

Twenty-one years later, two Canadian physicians made a discovery that could change the nature of medicine. Banting and Best developed a way of extracting insulin from the pancreas, thereby offering a means to treat what had, until then, been a rapidly fatal disease of childhood, type I diabetes [6]. For the first time, it became possible for people afflicted with a severe non-communicable disease to achieve a virtually normal lifespan. During those additional years they would be exposed to the same risks as anyone else, causing some to die prematurely from, for example, road traffic accidents or influenza. However, by the 1940s, it was clear that those with type I diabetes also faced a considerably elevated risk of a number of other health problems, affecting their vision, their cardiovascular system, their kidneys, and the peripheral nervous system among others. Slowly, these problems were recognised as the complications of diabetes, even though some of them, such as ischaemic heart disease, were also common in those without diabetes [7]. This poses a fundamental challenge for the epidemiologist. When someone with type I diabetes dies from a heart attack, is this because of the fact that they smoked heavily or is it because of their diabetes? And as diabetes affected so many body systems, some unfortunate individuals may suffer from damage to many organs. Which kills them? As if that wasn’t complex enough, the introduction of one after another life prolonging treatments, such as dialysis for kidney failure, keeps people alive as their diseases processes progressed, allowing time for complications of those treatments to emerge. Today, older patients may be living with perhaps eight or nine different medical conditions [8]. This is the situation that Goldberger and colleagues have sought to untangle. They were at least able to benefit from the fact that the names given to diseases are no longer arbitrary. However, as they show, the attribution of death to a single cause is still challenging, over a century since Farr noted how “in many cases the primary cause can, but in many cases it cannot be discovered” [9].

Given that many people with die with, or from, several conditions, how does one decide which is most important? Initially, countries made their own choices about how these decisions would be made, how many causes of death they would record on a death certificate, and how to allocate a main cause where several were present. A common structure was proposed at the Fifth Decennial Conference on the ICD, in 1938 but not adopted until 1948. This specified the immediate cause of death along with its antecedents, as well as other significant conditions contributing to the death but unrelated to the immediate cause and gave rise to the death certificate used today. The problem of attribution had long been recognised, including by Bertillon who had proposed some basic rules. However, the task was far from straightforward. By 1925 a Manual of Joint Causes of Death had been prepared, although with a caveat that it was “a temporary guide for those who are groping for help in making their assignments, rather than an authoritative manual”. This guidance was subsequently refined and incorporated within the International Classification of Diseases. However, no matter how precise any coding rules might be, they depend fundamentally on the accuracy and completeness of recording of causes of death.

## Accuracy of death certification and the use of autopsy

There are concerns about the accuracy of what is written on the death certificate everywhere. The diagnosis may simply be wrong or it may be vague, giving rise to the term “junk codes”, which although appearing in the ICD, are non-specific and of little help in identifying the underlying risk factors. In many countries, the problem is growing because of the marked reduction in the number of autopsies being conducted, in part because of the costs involved but also, in some cases, because of resistance by relatives of the deceased. Autopsies are permitted under all three of the Abrahamic religions represented in Israel, although in both Judaism [10] and Islam [11] there are certain aspects that continue to be debated and there is not unanimity among all of the branches of any of the three religions. Thus, autopsies are rejected by some ultra-orthodox Jewish groups, who succeeded in making Israeli law more restrictive in 1980. However, there is now a non-invasive virtual alternative, post-mortem computerised tomography scanning. This was first systematically compared with conventional autopsy in a 1994 study conducted in Israel [12]. The findings from conventional and virtual autopsy were compared, with the physicians involved being blind to the others’ findings. They concluded that both methods identified conditions missed by the other and, ideally, the two would be combined to maximise the yield of diagnostic information. Since then, considerable experience has been accumulated with a wide range of non-invasive and minimally invasive methods and a recent systematic review has concluded that the maximum diagnostic yield is achieved by combining standard computerised tomography, computerised tomographic angiography, and biopsies, but if micro-invasive methods cannot be used then computerised tomography should be combined with magnetic resonance imaging [13]. However, while these approaches offer important new possibilities, recent experience in Jerusalem, where virtual autopsies are conducted regularly, suggests that those opposed to conventional autopsy will also reject virtual autopsy [14]. Moreover, while real or virtual autopsies may clarify what conditions are present, it is often only the first step to deciding the cause of death.

## Multiple causes of death

The problems arising from multiple causes of death were first described in detail by Janssen, in the late 1930s in the United States [15]. He noted how 72 % of death certificates from New York contained more than one cause of death whereas the corresponding figure from Arizona was 35 %. Yet the differences were not simply geographical. As he noted, there was an “almost uniform lack of detail” on certificates for African-Americans. He also noted the considerable variation in the probability that a particular condition listed on the death certificate would be identified as the primary cause of death. Thus, there were twice as many death certificates mentioning alcoholism as those where it was listed as the primary cause. For pneumonia, the difference was threefold. Subsequent studies have shown how the probability of there being multiple causes on the death certificate varies according to the immediate cause, being low for cancer, for example [16].

In some cases this may not matter. Goldacre and colleagues have shown how, in over 96 % of death certificates where a cause of death was mentioned, acute myocardial infarction was recorded as the underlying cause and this did not change over a 10 year time period [17]. In other situations, such as studies of infant mortality [18] or cancer deaths [19] differences in which cause is selected as the main one leading to death can be important. However, what is essential is that it is at last considered in international comparisons of cause of death. Importantly, an increasing number of countries, including Israel, now produce mortality statistics that include multiple causes of death, thereby permitting more detailed analyses of the disease burden in the population and demographers working with these data are developing innovative methods and shared protocols [20]. Goldberger and colleagues review a number of papers from other countries showing the benefits of such analyses.

## Conclusions and further research

Mortality statistics are among the building blocks for health policy. One of the main goals of health policy is to reduce disease burden and, unless this can be quantified accurately, it is not possible either to set priorities or track progress. The paper by Goldberger and colleagues is an excellent practical example of what needs to be done. What is now needed are further international studies on the comparability of attribution of cause of death, building on the work described by Goldberger and colleagues, as well as studies of the effectiveness of interventions to improve the quality of coding. Finally, those engaged in health policy must be made aware of the caveats to be observed when making international comparisons of disease outcomes, something that they sometimes fail to do [21].

## References

[1] Goldberger N, Applbaum Y, Meron J, Hakai Z. High Israeli mortality rates from diabetes and renal failure - can international comparison of multiple causes of death reflect differences in choice of underlying cause? Israel J Health Pol Res. 2015.4:31. 10.1186/s13584-015-0027-6 PMC4590706 26430506

[2] Erhardt CL (1958). What is the cause of death?. J Am Med Assoc.

[3] Langmuir AD (1976). William Farr: founder of modern concepts of surveillance. Int J Epidemiol.

[4] Lewes FM (1988). Dr Marc d’Espine’s statistical nosology. Med Hist.

[5] Moriyama IM, Loy RM, Robb-Smith AHT, Rosenberg HMR, Hoyert DL (2010). History of the statistical classification of diseases and causes of death.

[6] Bliss M, Purkis R (1982). The discovery of insulin.

[7] Kessler II (1971). Mortality experience of diabetic patients: a twenty-six year follow-up study. Am J Med.

[8] Barnett K, Mercer SW, Norbury M, Watt G, Wyke S, Guthrie B (2012). Epidemiology of multimorbidity and implications for health care, research, and medical education: a cross-sectional study. Lancet.

[9] Humphreys NA (1885). Vital statistics: memorial volume of selections from the reports and writings of William Farr.

[10] Jakobovits I (1958). The dissection of the dead in Jewish Law: a comparative and historical study. Tradition.

[11] Mohammed M, Kharoshah MA (2014). Autopsy in Islam and current practice in Arab Muslim countries. J Forensic Legal Med.

[12] Donchin Y, Rivkind AI, Bar-Ziv J, Hiss J, Almog J, Drescher M (1994). Utility of postmortem computed tomography in trauma victims. J Trauma.

[13] Blokker BM, Wagensveld IM, Weustink AC, Oosterhuis JW, Hunink MG. Non-invasive or minimally invasive autopsy compared to conventional autopsy of suspected natural deaths in adults: a systematic review. Eur Radiol. 2015. doi:10.1007/s00330-015-3908-8. 10.1007/s00330-015-3908-8 PMC4778156 26210206

[14] Tal S, Berkovitz N, Gottlieb P, Zaitsev K (2015). Acceptance of forensic imaging in Israel. Isr Med Assoc J.

[15] Janssen TA (1940). Importance of tabulating multiple causes of death. Am J Public Health.

[16] Mackenbach JP, Kunst AE, Lautenbach H, Bijlsma F, Oei YB (1995). Competing causes of death: an analysis using multiple-cause-of-death data from The Netherlands. Am J Epidemiol.

[17] Goldacre MJ, Roberts SE, Griffith M (2003). Multiple-cause coding of death from myocardial infarction: population-based study of trends in death certificate data. J Public Health Med.

[18] Nam CB, Eberstein IW, Deeb LC, Terrie EW (1989). Infant mortality by cause: a comparison of underlying and multiple cause designations. Eur J Popul.

[19] Manton KG, Wrigley JM, Cohen HJ, Woodbury MA (1991). Cancer mortality, aging, and patterns of comorbidity in the United States: 1968 to 1986. J Gerontol.

[20] Désesquelles AF, Salvatore MA, Pappagallo M, Frova L, Pace M, Meslé F (2012). Analysing multiple causes of death: Which methods for which data? An application to the cancer-related mortality in France and Italy. Eur J Popul.

[21] Spiegelhalter D (2013). Are you 45 % more likely to die in a UK hospital rather than a US hospital?. BMJ.

